# Author’s response

**DOI:** 10.1308/003588413X13511609956615

**Published:** 2013-01

**Authors:** J Wasson

**Affiliations:** Peterborough and Stamford Hospitals NHS Foundation Trust,UK

While our paper confirms the obvious assumption that caliper measurement is more accurate than clinical palpation, it also highlights that caliper measurement is statistically comparable with accurate ultrasonography measurement for clinically palpable neck lumps. We therefore emphasise the merit of this inexpensive adjunct in assessing neck lump size when more expensive tools are not immediately available.

Our study highlights the use of calipers in augmenting clinical assessment at neck lump clinics. As previously discussed, we acknowledge that calipers cannot substitute ultrasonography in the assessment of lump morphology, vascular flow and anatomical origin or targeting for fine needle aspiration. We also appreciate that different nodal levels have varying acceptable sizes for normality. Suspicious lymph nodes with a minimal axial diameter greater than 10mm (15mm for junctional nodes) have a sensitivity and specificity of approximately 70% for neoplastic involvement.^1^ For this reason, all neck lumps greater than 9mm in size are selected for ultrasonography assessment in addition to smaller neck lumps with a high index of clinical suspicion for neoplastic involvement.

All data were obtained in an ear, nose and throat neck lump clinic. This is why oral and maxillofacial surgeons were not mentioned in the paper. However, we fully acknowledge that head and neck cancer management is a multidisciplinary effort to which oral and maxillofacial surgeons provide an invaluable contribution.
